# Is Complete Re-Osseointegration of an Infected Dental Implant Possible? Histologic Results of a Dog Study: A Short Communication

**DOI:** 10.3390/jcm9010235

**Published:** 2020-01-16

**Authors:** Markus Schlee, Loubna Naili, Florian Rathe, Urs Brodbeck, Holger Zipprich

**Affiliations:** 1 Private Practice, Forchheim, Germany; markus.schlee@32schoenezaehne.de (M.S.); florian.rathe@32schoenezaehne.de (F.R.); 2 Department of Maxillofacial Surgery, Goethe University, 60590 Frankfurt am Main, Germany; 3 Private Practice, Kirchbachstraße 186, 28211 Bremen, Germany; lnaili@students.uni-mainz.de; 4 Private Practice and Department of Prosthodontics, Danube University, 3500 Krems, Austria; 5 Private Practice, 8051 Zürich, Switzerland; ursbrodbeck@bluewin.ch; 6 Department of Prosthodontics, Goethe University, 60590 Frankfurt am Main, Germany

**Keywords:** periimplantitis, electrolytic cleaning, augmentation, air flow, re-osseointegration, dog study

## Abstract

Complete reosseointegration after treatment of periimplantitis was never published yet. This short scientific communication reports about results of a randomized controlled preclinical study. An electrolytic approach was compared to a classical modality (ablative, cotton pellets soaked with sodium chloride solution and H_2_O_2_. For electrolytic cleaning a complete reosseointegration was achieved in several cases serving as a proof of concept.

## 1. Introduction

Ligature-induced inflammation and bone loss in a dog model was introduced to imitate periimplantitis in humans and preclinically study different treatment approaches. Schwarz et al. [1] compared bone defects in 24 patients with advanced periimplantitis to ligature-induced bone defects at 30 implants in five beagle dogs. Defect morphology was similar in both entities. The authors concluded that the dog model would be an appropriate model for simulating periimplantitis in humans. Seong at al. reported the possibility of spontaneous healing after ligature removal in dogs [2]. The authors claimed that further clarification of differences between the forced acute bone loss induced by ligatures and the chronic process in clinical periimplantitis would be necessary. Nevertheless, the dog model provided valuable insights into the etiology and pathogenesis of periimplantitis [3] and may be a reasonable model for studying whether re-osseointegration of implant surfaces covered by bacterial biofilms is possible. Based on animal studies, re-osseointegration is feasible, although unpredictable, as it varies between studies and depends on implant surface characteristics and defect morphology [4,5].

In this preclinical randomized and controlled trial, electrolytic cleaning (EC) versus classical cleaning (CC, rubbing a gauze strip soaked in H_2_O_2_ (3%), followed by a strip soaked in saline (0.9%)) was employed to prove whether complete re-osseointegration is possible in a dog model. This approach was chosen on the basis of previous studies [5]. A custom-made pilot series device and the pilot electrolyte (sodium and potassium iodine) of the in vitro study of Ratka et al. [6] were used in this study. The aim of this short scientific communication is to share histologic results and to prove whether complete re-osseointegration is possible when EC has been used as a decontamination technique.

## 2. Experimental Section

**Figure 1:**
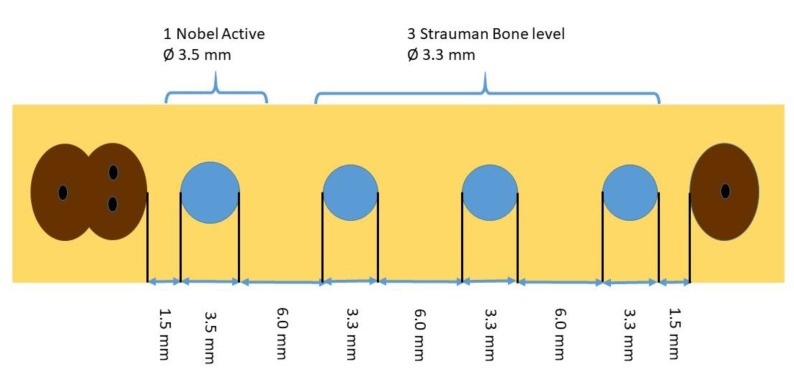
Allocation of implant positions.

**Figure 2:**
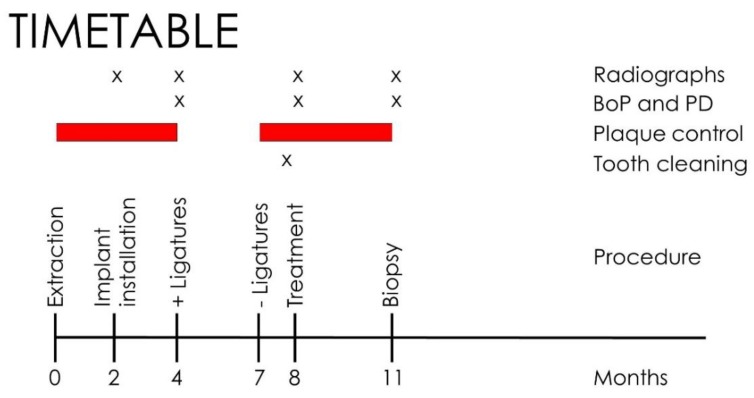
Timetable of the dog study.

The study was approved by the Internal Review Board of the University of São Paulo (2013.1.1193.58.3 by CEUA (Comissão de Ética no Uso de Animais)). No sample size calculation could be done because this was the first in vivo trial and no data were available. To have a high chance of achieving significance, we used a large number of dogs compared to existing literature (8 dogs). Two months after extraction (T1) of all premolars and the first molars in the left and right mandible, 4 implants per quadrant were placed (Figure 1). It was planned to place two different brands of implants: 48 BL implants (BL SLA^®^ 3.3 × 8.0 mm, Straumann, Basel, Switzerland) and 16 Nobel Active implants (NA TiUnite^®^ 3.5 × 8.5mm, Nobel Biocare, Kloten, Switzerland). In view of the expected bone defects, the inter-implant distance was at least 6 mm (Figure 1). In addition to the study implants, extra implants were placed, provided there was sufficient space and the study implants would not be impeded. Ligatures were pushed into the sulcus at T2 (4 months) and removed at T3 (7 months). A hygiene program was set up, and the implants were treated at T4 (8 months) by either CC or EC in a randomized manner with the use of sealed envelopes. The mode of action of EC has been described before [6,7]. In summary, the implants were negatively loaded with a current of around 5 V, and rinsed with an electrolyte which passed an anode inside a spray-head (Figure 4c). Sodium iodine (200 g/L), potassium iodine (200 g/L), and L (+)-lactic acid (20 g/L) were used as the electrolyte [6] and an early prototype of a spray-head (Figure 4c) as the source of current (5 min, 400 mA) in the present study. The current directs anions and cations to the electric field. While the anions at the anode form an iodine disinfecting solution, the cations penetrate the biofilm, take an electron from the implant surface, and the emerging hydrogen bubbles (H_2_) lift off the bacterial biofilm. The CC implants were treated by rubbing a gauze strip soaked in H_2_O_2_ (3%), followed by a strip soaked in saline (0.9%, Braun Melsungen, Germany). All sites were augmented with autogenous bone harvested from the ramus area of the lateral mandible (Micross Safescraper, Zantomed, Duisburg, Germany), covered with a collagen membrane (Bio-Gide, Geistlich, Wohlhusen, Switzerland), and the flap was coronally advanced to cover the augmented area without tension. One implant per dog (2 Nobel, 4 Straumann) was randomly selected and not treated to serve as a negative control. The animals were sacrificed for histologic evaluation at T5 (11 months). Standardized x-rays were taken at T1, 2, 4, and 5. Bleeding on probing (BoP) as well as an assessment of pocket depth (PD) at T2, 4, and 5 (Figure 2) were performed. Preparation of samples followed the technique introduced by Donath [8], and staining was done with Stevenel’s blue or Toluidine blue. Bone gain was assessed using a digital tool (NDP.view 2.5.19, (Hamamatsu Photonics KK, Shizuoka, Japan)) and analyzed histomorphometrically.

## 3. Results

**Figure 3:**
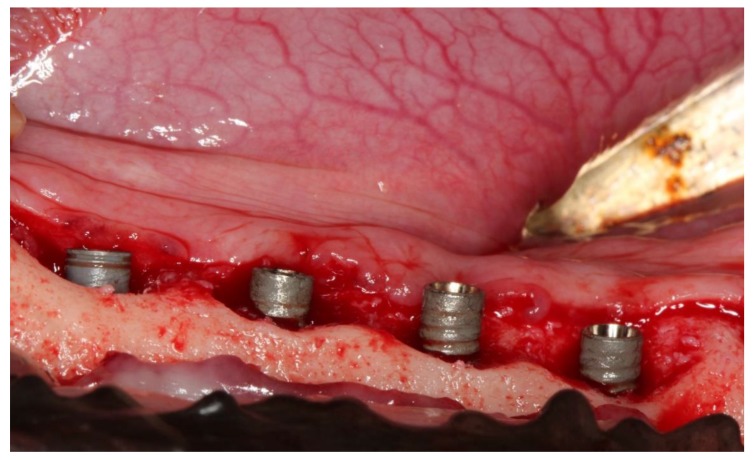
Massive horizontal bone loss results in merging bone defects and massive resorption of the whole ridge.

**Figure 4:**
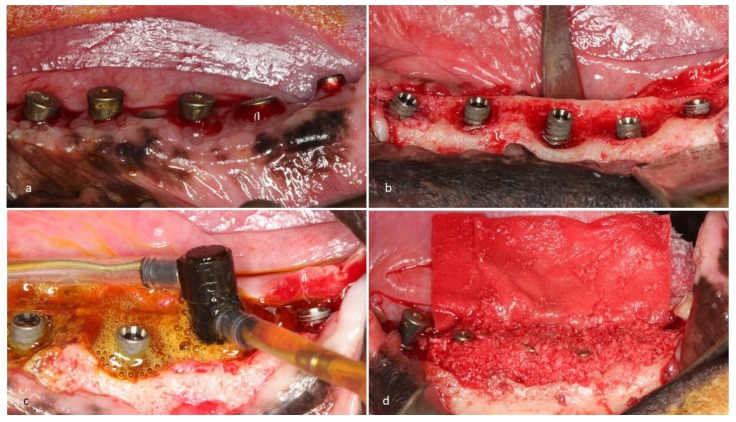
(**a**) Infected site prior to treatment, (**b**) reflected flap indicates a complex merged defect morphology, (**c**) electrolytic cleaning with a spray-head, (**d**) augmented site before collagen membrane was applied.

**Figure 5:**
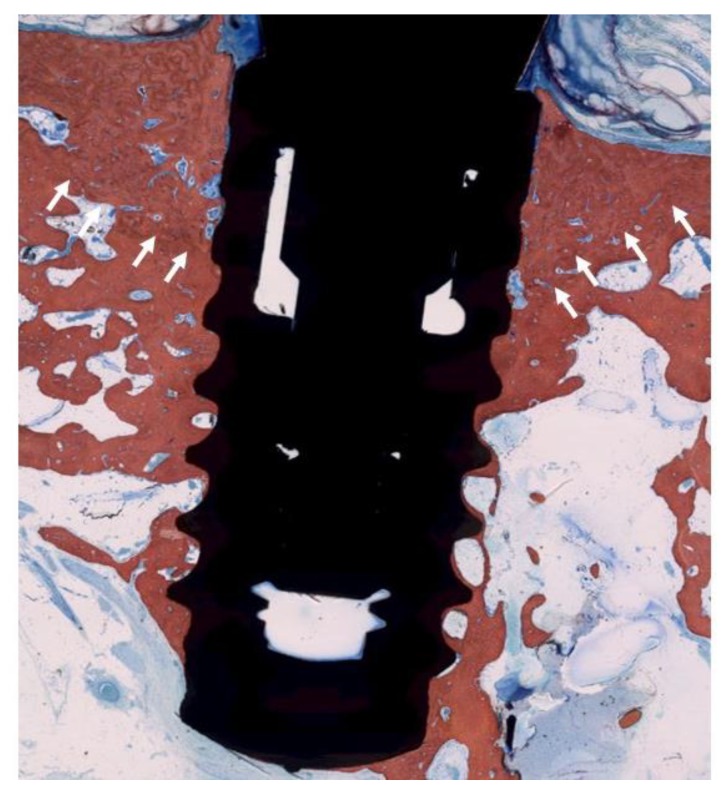
Histologic evaluation of healing after electrolytic cleaning (EC) (SLA^®^ surface). The arrows indicate the bony crest before treatment. Complete coverage of the formerly exposed implant surface was achieved.

**Figure 6:**
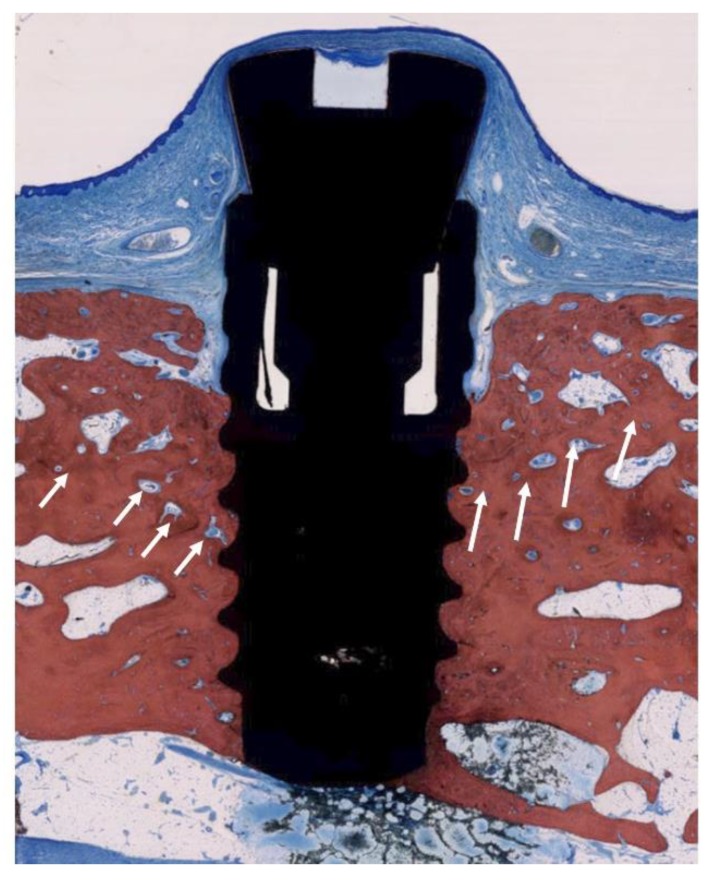
Histologic evaluation of healing after classical cleaning (CC) (SLA^®^ surface). The arrows indicate the bony crest before treatment. Bone-to-implant contact was achieved but full regeneration could not be achieved in any implant.

**Figure 7:**
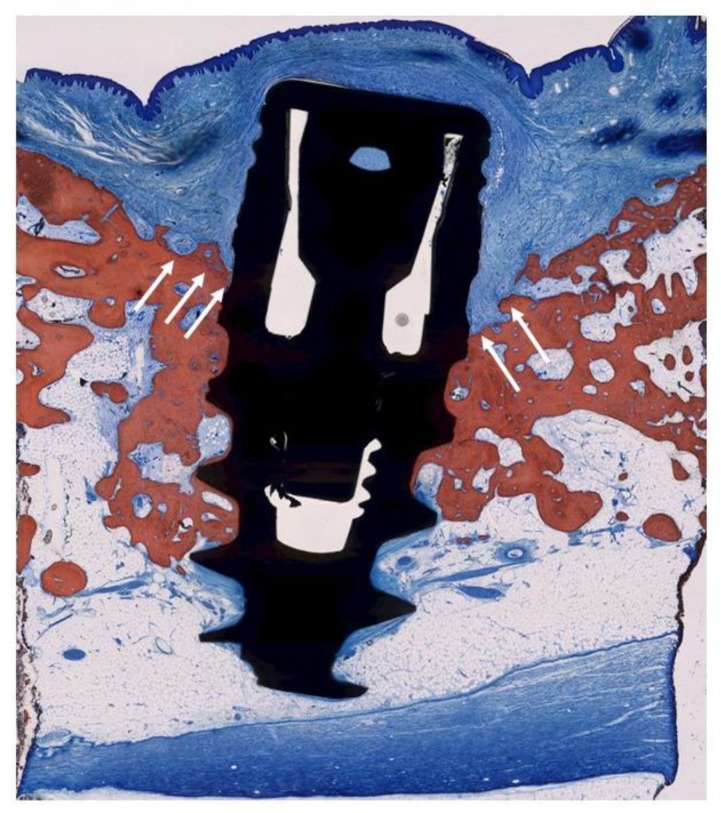
Histologic evaluation of healing after CC (TiUnite^®^ surface). The arrows indicate the bony crest before treatment. A small amount of new bone-to-implant contact was achieved. No re-osseointegration could be achieved on major parts of the implant surface.

**Figure 8:**
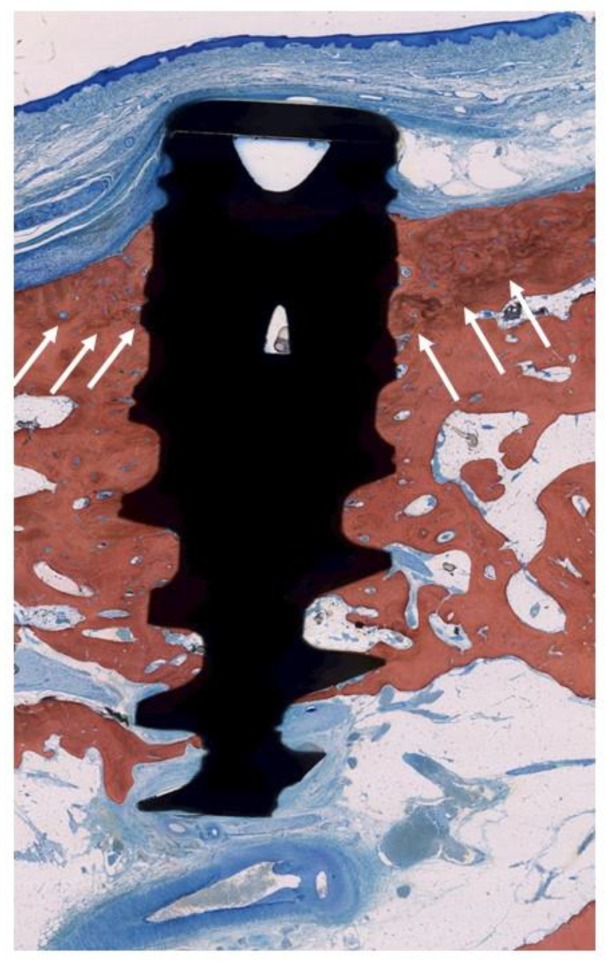
Histologic evaluation of healing after EC (TiUnite^®^ surface). The arrows indicate the bony crest before treatment. Bone-to-implant contact was achieved as more crestal than the neighboring bone level.

**Figure 9:**
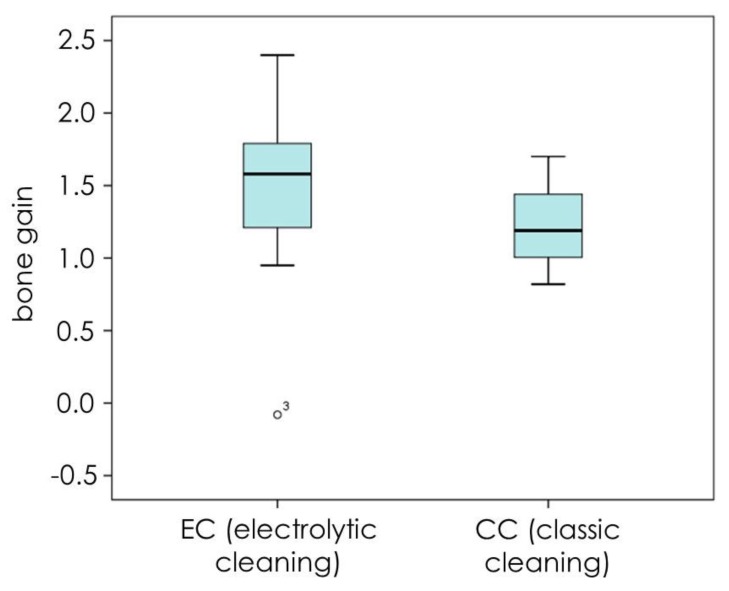
Bone gain for submerged implants at T5 in CC (8 implants) and EC (9 implants) groups display higher bone gain for the EC group.

Sixty-five implants were finally placed (Table 1). Fourteen TiUnite^®^ implants and 43 SLA^®^ implants were treated (EC & CC) and eight implants (2 TiUnite^®^ and 6 SLA^®^) were left untreated. Two implants could not be placed due to the required distances (Figure 1). All implants displayed massive horizontal bone loss before treatment, in most cases merging with neighboring defects (Figure 3 and Figure 4), which left a site that was difficult to augment. Forty-eight implants were exposed during healing whereas nine EC (5 TiUnite^®^ and 4 SLA^®^) and eight CC (3 TiUnite^®^ and 5 SLA^®^) implants stayed covered until T5. Complete re-osseointegration could be demonstrated for implants treated by CC in one implant and for four TiUnite^®^ and six SLA^®^ implants treated by EC (Figure 5, Figure 6, Figure 7 and Figure 8). The median bone gain for implants which healed submerged was 1.58 ± 0.76 in the case of EC and 1.19 ± 0.29 in the case of CC. The boxplot appears in Figure 9. 

## 4. Discussion

The results of this preclinical study may differ from results gained with the final devices used for this technique. Nevertheless, they may serve as proof of principle. As submerged healing was sought, the high number of exposures was an undesirable result. This may correlate with the fact that most of the defects were horizontal and required vertical augmentation. Furthermore, uneventful healing was not possible because of dogs biting on the surgical sites. Implants exposed to the platform cannot regenerate completely and were therefore excluded from statistical evaluation. This limits the meaningfulness of the study. Consequently, we were not able to prove the superiority of one of the treatment modalities in regenerating bone in contact with the implant surface because of the sample size. 

Nevertheless, 17 implants were not exposed. When comparing bone regeneration in EC and CC in these cases, EC tended to be superior based on median, without reaching significance. Eight implants in the EC group achieved complete re-osseointegration, but none in the CC group. To the best of the authors’ knowledge, complete regeneration has never before been described in the literature. This paper does not investigate the reasons for this observation. In the context of previous literature, it may be assumed that EC in contrast to CC removes the bacterial biofilm and other remnants to an extent that makes complete re-osseointegration possible [6,7]. The latter was proven through histology (Figure 5, Figure 6, Figure 7 and Figure 8). Re-osseointegration is defined as new bone in direct contact with the formerly infected implant surface. Soft tissue thickness correlates to bone level. Thus bone regeneration to the platform cannot be expected in the case of exposure if the shoulder of the implant was visible [9]. If chewing forces were applied to the implant, complete regeneration also cannot be expected, even if the site stayed covered. Interestingly, complete regeneration was only possible in the EC group—even under these circumstances. 

The regenerative potential of horizontal bone defects with no walls is worse than for contained, multi-walled defects [4,7]. In this animal study we observed massive horizontal bone loss and merging defects resulting in a severely resorbed ridge (Figure 3). In humans, an inter-implant distance of 3 mm is usually sufficient to maintain an interdental bone peak in the case of bone loss around implants [10]. We used an inter-implant distance of 6 mm and yet the defects still merged. This might be an indication that ligature-induced periimplantitis in a dog model behaves differently to periimplantitis in humans. Considering our findings, dog models might be inappropriate for investigating the amount of bone generation. Fewer implants per ridge are suggested for future dog studies. 

## 5. Conclusions

As a proof of concept, complete re-osseointegration was proven after electrolytic cleaning and augmentation.

## Figures and Tables

**Table 1:** Allocation of implant positions.

Dog	Quadrant	Straumann	Nobel	Straumann Not Treated	Nobel Not Treated	Total	Not Placed
1	right	2	1			3	1
	left	3	1	1		5	
2	right	3	1			4	
	left	3	1	1		5	
3	right	3	1			4	
	left	2	1	1		4	
4	right	3	0		1	4	
	left	3	1			4	
5	right	3	1			4	
	left	3	0		1	4	
6	right	3	1	1		5	
	left	3	1			4	
7	right	2	1			3	1
	left	2	1	1		4	
8	right	3	1			4	
	left	2	1	1		4	
total		43	14	6	2	65	2
Straumann						49	
Nobel						16	

## References

[1] Schwarz F., Herten M., Sager M., Bieling K., Sculean A., Becker J. (2007). Comparison of naturally occurring and ligature-induced peri-implantitis bone defects in humans and dogs. Clin. Oral Implants Res..

[2] Seong W.J., Kotsakis G., Huh J.-K., Jeong S.C., Nam K.Y., Kim J.R., Heo Y.C., Kim H.-C., Zhang L., Evans M.D. (2019). Clinical and microbiologic investigation of an expedited peri-implantitis dog model: An animal study. BMC Oral Health.

[3] Schwarz F., Sculean A., Engebretson S.P., Becker J., Sager M. (2015). Animal models for peri-implant mucositis and peri-implantitis. Periodontol. 2000.

[4] Schwarz F., Sahm N., Schwarz K., Becker J. (2010). Impact of defect configuration on the clinical outcome following surgical regenerative therapy of peri-implantitis. J. Clin. Periodontol..

[5] Renvert S., Polyzois I., Maguire R. (2009). Re-osseointegration on previously contaminated surfaces: A systematic review. Clin. Oral Implants Res..

[6] Ratka C., Weigl P., Henrich D., Koch F., Schlee M., Zipprich H. (2019). The Effect of In Vitro Electrolytic Cleaning on Biofilm-Contaminated Implant Surfaces. J. Clin. Med..

[7] Schlee M., Rathe F., Brodbeck U., Ratka C., Zipprich H. (2019). Treatment of periimplantitis-electrolytic cleaning versus mechanical and electrolytic cleaning—A randomized controlled clinical trial—6 month results. J. Clin. Med..

[8] Jovanovic S.A., Kenney E.B., Carranza F.A., Donath K. (1993). The regenerative potential of plaque-induced peri-implant bone defects treated by a submerged membrane technique: An experimental study. Int. J. Oral Maxillofac Implants.

[9] Linkevicius T., Puisys A., Linkeviciene L., Peciuliene V., Schlee M. (2015). Crestal Bone Stability around Implants with Horizontally Matching Connection after Soft Tissue Thickening: A Prospective Clinical Trial. Clin. Implant Dent. Relat. Res..

[10] Tarnow D.P., Cho S.C., Wallace S.S. (2000). The effect of inter-implant distance on the height of inter-implant bone crest. J. Periodontol..

